# Early-pregnancy BMI-derived adiposity change rate and gestational diabetes mellitus risk: modification by a fatty liver index–defined hepatic-metabolic phenotype

**DOI:** 10.3389/fendo.2026.1889628

**Published:** 2026-09-15

**Authors:** Jing Zhao, Yingjie Chen, Mengjia Peng, Binbin Shao, Jian Zhu, Hong Cao

**Affiliations:** 1 Department of Endocrinology, Affiliated Hospital of Jiangnan University, Jiangnan University, Wuxi, China; 2 School of Biotechnology, Jiangnan University, Wuxi, China; 3 Department of Emergency, Shishi Municipal Hospital, Shishi, China; 4 Department of Gynecology and Obstetrics, The Third Affiliated Hospital of Wenzhou Medical University, Rui’an, China

**Keywords:** adiposity change, early pregnancy, estimated body fat percentage, fatty liver index, gestational diabetes mellitus, hepatic-metabolic phenotype, risk stratification

## Abstract

Gestational diabetes mellitus (GDM) is commonly diagnosed at 24–28 weeks of gestation; however, metabolic susceptibility that precedes dysglycemia may emerge earlier. We examined whether the rate of early pregnancy body mass index (BMI)-derived estimated body fat percentage (eBFP) change was associated with subsequent GDM, and whether this association differed according to an early hepatic–metabolic phenotype defined by the natural log-transformed fatty liver index (FLI_Ln). This secondary analysis used de-identified data from a prospective Korean pregnancy cohort and included 583 singleton pregnant women without pregestational diabetes, of whom 37 developed GDM. eBFP was estimated from BMI and maternal age, and the early-pregnancy eBFP change rate was calculated from pre-pregnancy to 10–14 weeks of gestation and scaled so that each one-unit increase represented a 0.1 percentage-point/week higher change rate. Multivariate logistic regression, restricted cubic splines, threshold analysis, subgroup interaction testing, exploratory mediation analysis, and propensity score–matched sensitivity analyses were also performed. After adjustment for maternal age, parity, pre-pregnancy BMI, a higher eBFP change rate was associated with increased odds of subsequent GDM (odds ratio [OR], 1.58; 95% confidence interval [CI], 1.14–2.21; P = 0.006), with an approximately linear dose–response pattern. The FLI_Ln showed a nonlinear association with GDM, with a data-derived threshold of 2.90. The eBFP change rate–GDM association was evident among women with FLI_Ln ≥2.90 (OR, 1.82; 95% CI, 1.22–2.71; P = 0.004), but not among those with FLI_Ln <2.90 (OR, 0.51; 95% CI, 0.22–1.20; P = 0.123; P for interaction = 0.018). Exploratory mediation analyses suggested that FLI_Ln partially accounted for the observed association, although the magnitude of this mediation was imprecisely estimated. In this secondary cohort analysis, a faster early-pregnancy BMI-derived adiposity change was associated with a higher risk of GDM, particularly among women with an elevated composite hepatic–metabolic phenotype. These findings support an early adiposity–hepatic metabolic susceptibility framework for GDM risk enrichment but require external validation before clinical application.

## 1 Introduction

Gestational diabetes mellitus (GDM) affects approximately one in six pregnancies globally (1). Beyond immediate obstetric implications, it confers long-term cardiometabolic risks to both mother and offspring, including future type 2 diabetes and cardiovascular disease (2, 3). In routine practice, however, GDM screening is not performed until 24–28 weeks of gestation (4). This creates a temporal gap: metabolic susceptibility may precede detectable dysglycemia by weeks or months. Early-pregnancy markers that identify at-risk women before mid-pregnancy screening could therefore enable earlier risk stratification and inform targeted prevention strategies.

Pre-pregnancy body mass index (BMI) is a well-established risk factor for GDM, but it reflects baseline maternal body size rather than early gestational change in adiposity-related status (5). Women with comparable pre-pregnancy BMI may follow divergent adiposity trajectories after conception, as maternal energy storage and substrate partitioning begin to shift during early pregnancy (6, 7) — well before conventional GDM screening is performed. We operationalized this dynamic marker as the gestational-age-standardized change in BMI-derived estimated body fat percentage (eBFP) from pre-pregnancy to 10–14 weeks of gestation, hereafter referred to as the eBFP change rate. Because eBFP was derived from BMI and maternal age rather than from direct body-composition techniques. Therefore, the eBFP change rate should be interpreted as a scalable BMI-derived proxy for early adiposity-related change, not as a direct measure of fat-mass gain or as an independent measure of BMI change. Nonetheless, given that direct body-composition assessment is seldom feasible in routine antenatal care, a BMI-derived measure offers a pragmatic compromise. Evaluating this marker after adjustment for maternal age and pre-pregnancy BMI may help determine whether early gestational change provides risk information beyond that explained by baseline maternal body size.

Early hepatic metabolic status may provide critical context for interpreting how adiposity-related changes translate into GDM risk. Hepatic metabolic dysfunction in early pregnancy, including NAFLD diagnosed at 10–14 weeks, has been associated with subsequent GDM (8, 9). Recent work further shows that first-trimester noninvasive indices, including the hepatic steatosis index (HSI) and fatty liver index (FLI), predict subsequent GDM (10, 11). Together, these findings suggest that early liver metabolic status is not simply a confounder to adjust for, but a metabolic milieu within which adiposity changes may become diabetogenic. We therefore use the natural log-transformed FLI (FLI_Ln) to characterize this hepatic-metabolic context. It should be noted that because FLI incorporates BMI, waist circumference (WC), triglycerides (TG), and γ-glutamyl transferase (GGT), FLI_Ln is best understood as a clinically accessible composite phenotype reflecting adiposity, dyslipidemia, and hepatic enzymatic activity — not as a liver-specific biomarker (12). Accordingly, we examined whether the eBFP change rate–GDM association differed according to FLI_Ln-defined hepatic-metabolic status.

Despite extensive prior work on pre-pregnancy BMI, NAFLD, and hepatic steatosis indices as independent GDM predictors (5, 8–12), three interrelated knowledge gaps remain: First, existing risk stratification relies predominantly on static pre-pregnancy BMI and does not capture dynamic early-gestational adiposity changes that may identify additional at-risk women. Second, the potential for early-pregnancy hepatic-metabolic phenotypes (e.g., FLI_Ln) to serve as effect modifiers in the adiposity–GDM pathway has not been examined. Third, no prior study has jointly modeled the rate of change in early-pregnancy adiposity and an FLI-defined hepatic-metabolic phenotype to assess their combined contribution to GDM risk stratification. Addressing these gaps could shift early GDM risk assessment from a static obesity classification to a dynamic model of metabolic susceptibility, with actionable implications for targeted monitoring or lifestyle interventions before routine mid-pregnancy screening. To address these gaps, we analyzed prospective cohort data (10–14 weeks of gestation) to: (i) test whether the eBFP change rate predicts subsequent GDM after adjustment for pre-pregnancy BMI; (ii) evaluate whether FLI_Ln statistically mediates part of this association; and (iii) determine whether elevated FLI_Ln defines a susceptible subgroup in which the adiposity–GDM link is amplified. We hypothesized that a higher eBFP change rate would be associated with an increased risk of GDM, particularly among women with elevated FLI_Ln.

## 2 Methods

### 2.1 Study design, data source, and ethics

This study was a secondary analysis of de-identified, publicly available data from the “Fatty Liver in Pregnancy” registry, a prospective Korean pregnancy cohort registered at ClinicalTrials.gov (NCT02276144) (8, 9). The registry enrolled women with singleton pregnancies who attended prenatal care before 14 weeks of gestation at two Korean centers and was designed to evaluate NAFLD and related metabolic phenotypes during pregnancy (8, 9). The analytic dataset for the present secondary analysis was obtained from the Supporting Information accompanying the article by Lee et al., in which the de-identified individual-level data were made publicly available (8).

The original cohort study was approved by the relevant institutional review board, and written informed consent was obtained before enrollment (8, 9). Additional consent was not required because this analysis used only de-identified public data and involved no participant contact, biological sample collection, or intervention. The present study adhered to the Declaration of Helsinki and reported according to the Strengthening the Reporting of Observational Studies in Epidemiology (STROBE) statement (13, 14).

### 2.2 Study population

The registry enrolled 663 singleton pregnant women without pregestational diabetes, heavy alcohol consumption, or chronic liver disease. Of them, 623 remained after excluding women who were lost to follow-up or delivered before 34 weeks (8). We further excluded participants with missing GDM status (n = 13), pre-pregnancy BMI (n = 1), HSI (n = 22), FLI or its components (n = 3), and gestational age at early-pregnancy assessment (n = 1). The final cohort included 583 women, of whom 37 developed GDM. The participant selection process is illustrated in **Figure 1**.

**Figure 1:**
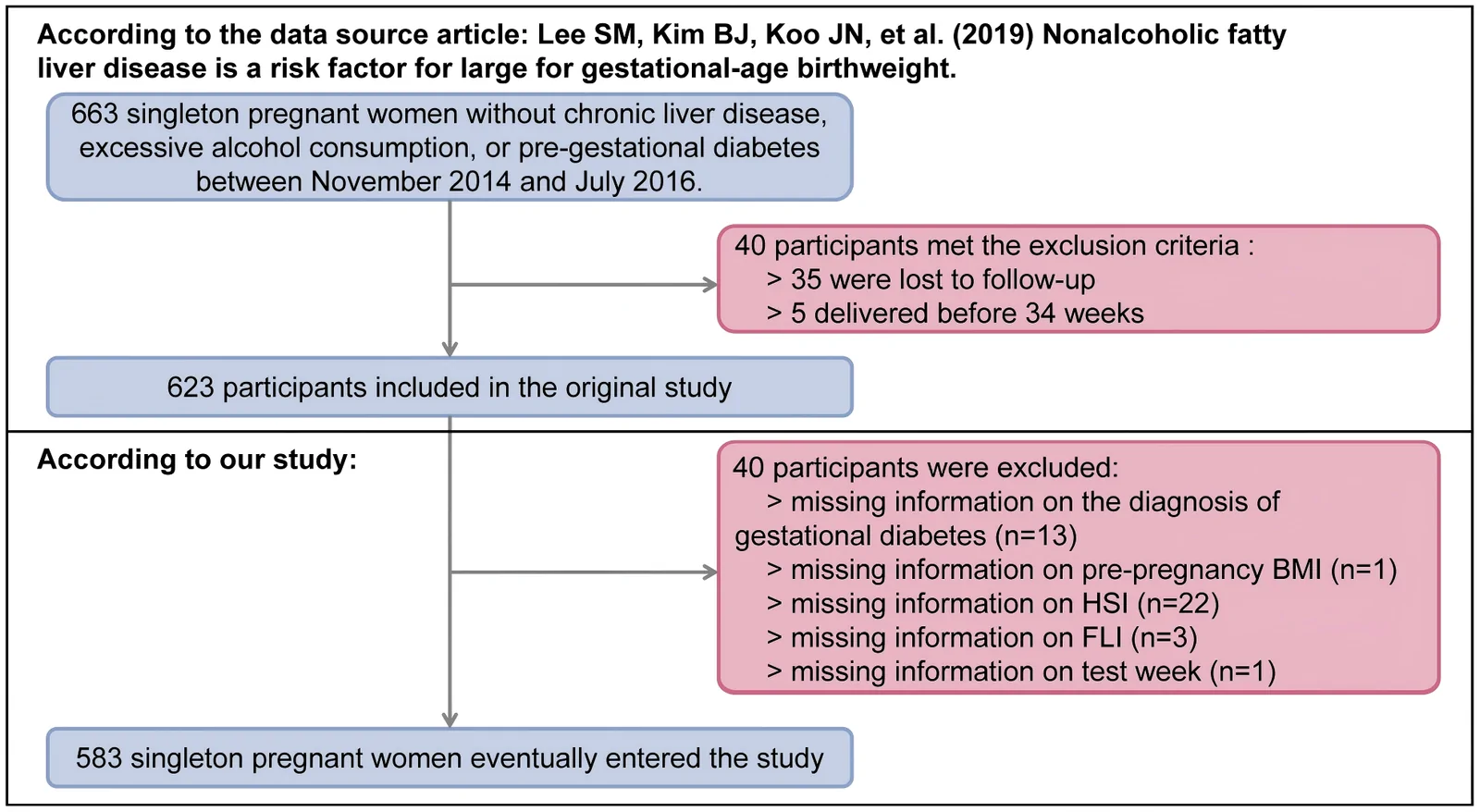
Flow diagram of participant selection for the analytic cohort.

### 2.3 Data collection

Data were collected as described previously (8, 9). The baseline demographic, obstetric, and clinical data were collected using structured questionnaires. At 10–14 weeks, participants underwent anthropometric assessment, liver ultrasonography, and fasting blood sampling. Stored liver ultrasound images were reviewed by experienced radiologists blinded to the clinical and biochemical data (8, 9). Laboratory measurements included liver and metabolic markers (8, 9). Additionally, we extracted the maternal age, parity, pre-pregnancy BMI, HSI, alanine aminotransferase (ALT), aspartate aminotransferase (AST), FLI, and gestational age at assessment for this secondary analysis.

### 2.4 Variables

#### 2.4.1 Exposure variable

The primary exposure was the eBFP change rate. The eBFP was calculated from the BMI and maternal age using the following validated equation because direct body composition data were unavailable (7):


estimated BFP(%) = 1.20 × BMI + 0.23 × age − 5.4.

Pre-pregnancy eBFP was calculated from pre-pregnancy BMI, whereas early-pregnancy BMI was back-calculated from the HSI formula because it was not available in the dataset (15). Because all participants were women and those with pregestational diabetes were excluded, the HSI formula was applied with the female coefficient retained and the diabetes coefficient omitted:


HSI = 8 × ALT/AST + BMI + 2;

therefore,


BMI10–14w = HSI − 8 × ALT/AST − 2.

Because this back-calculation is an algebraic identity rather than a model-based estimation, it introduces no additional estimation error; however, the precision of the recovered BMI is limited by the reporting precision of the deposited HSI and ALT/AST values. The eBFP at 10–14 weeks of gestation was calculated using the same equation. The eBFP change rate was defined as follows:


eBFP_dif_mean=(eBFP10–14w−pre‐pregnancy eBFP)/gestational age at assessment

This value was multiplied by 10 and analyzed as eBFP_dif_mean10; a 1-unit increase represented a 0.1 percentage-point/week increase in the eBFP change rate.

#### 2.4.2 Outcome variable

The primary outcome was subsequent GDM. In the original cohort, GDM was assessed at 24–28 weeks using a two-step strategy (9, 16). First, a non-fasting 50g glucose challenge test was performed, for which 1-h glucose level ≥ 7.8 mmol/L was considered positive. Subsequently, screen-positive women underwent a 100g oral glucose tolerance test (OGTT). GDM was diagnosed when at least two values met or exceeded Carpenter–Coustan thresholds: fasting ≥ 5.3, 1-h ≥ 10.0, 2-h ≥ 8.6, or 3-h ≥ 7.8 mmol/L (9, 16).

#### 2.4.3 Covariates and liver-related metabolic variables

Covariates were selected *a priori*. The main covariates were maternal age, parity, pre-pregnancy BMI, and gestational age at the time of assessment. Data regarding the FLI was available in the public dataset, and it was calculated using the BMI, waist circumference (WC), triglycerides (TG), and γ-glutamyl transferase (GGT) as follows (12):


FLI = exp(ModelFLI)/[1 + exp(ModelFLI)] × 100,

where


ModelFLI = 0.953 × ln(TG) + 0.139 × BMI + 0.718 × ln(GGT) + 0.053 × WC − 15.745.

As the FLI was right-skewed, it was naturally log-transformed and denoted as FLI_Ln. Given that the FLI incorporates BMI, WC, TG, and GGT, FLI_Ln was interpreted as a composite liver-related metabolic index rather than a liver-specific biomarker. This index was used for threshold identification, stratification, interaction testing, and exploratory explanatory analyses, but not as a routine covariate in the main models.

### 2.5 Statistical analysis

The final cohort analyzed included only complete-case data. As this was a secondary analysis, no *a priori* power calculations were performed. Continuous variables are summarized as mean ± standard deviation (SD) or median (interquartile range), while categorical variables are summarized as n (%). Data was compared between groups using the Student’s t test, Mann–Whitney U test, χ² test, or Fisher’s exact test, as appropriate.

The primary analysis of the present secondary study used multivariable logistic regression to estimate the association between the scaled early-pregnancy eBFP change rate (eBFP_dif_mean10) and subsequent GDM. The results are reported as odds ratios (ORs) and 95% confidence intervals (CIs). The primary model was adjusted for maternal age, parity, and pre-pregnancy BMI. Given the limited number of events, the models were pre-specified and parsimonious. Collinearity was assessed using variance inflation factor (VIF) analysis, with VIF < 5 considered acceptable (17). Receiver operating characteristic (ROC) curves were generated to evaluate discriminatory ability, and AUCs were compared using DeLong’s test.

Restricted cubic spline models were fitted to evaluate potential nonlinear associations between each continuous predictor and its corresponding outcome: eBFP change rate with GDM, FLI_Ln with GDM, and eBFP change rate with FLI_Ln. For each spline model, four knots were placed at the 5th, 35th, 65th, and 95th percentiles, respectively, of the corresponding continuous predictor distribution (18). Logistic regression was used for GDM outcomes, and linear regression was used for FLI_Ln outcomes. Likelihood ratio tests were used to assess overall and nonlinear associations. The spline findings were considered exploratory. Segmented regression was used for the exploratory FLI_Ln threshold analysis (19). The threshold FLI_Ln = 2.90 was treated as a data-derived risk-stratification cutoff, rather than a validated clinical diagnostic threshold.

Subgroup analyses were conducted according to maternal age, parity, pre-pregnancy BMI (< 23 vs. ≥ 23 kg/m²), and FLI_Ln status (< 2.90 vs. ≥ 2.90) (20, 21). Interactions were assessed using product terms, and models omitted the stratification variable when applicable. Stratified splines were used only when an interaction was statistically significant.

Exploratory explanatory analyses were performed to assess whether FLI_Ln might partly account for the association between eBFP change rate and GDM. Mediation analyses were performed using elevated FLI_Ln (≥ 2.90) and continuous FLI_Ln as mediators, with 1,000 bootstrap repetitions using the mediation package (22). As the eBFP change rate and FLI_Ln share BMI-related components, these analyses were treated as exploratory statistical decomposition and were not interpreted as causal mediation.

Supportive analyses included joint visualization of the continuous eBFP change rate and FLI_Ln using logistic generalized linear models with natural cubic splines and 1:1 nearest-neighbor propensity score matching by FLI_Ln status. Matching was performed with a 0.02 caliper width based on maternal age, parity, pre-pregnancy BMI, and gestational age at assessment. The standardized mean differences (SMD) < 0.10 indicated an acceptable balance (23).

All analyses were performed using R 4.2.2 and Free Statistics 2.3. Tests were two-sided, with P < 0.05 considered significant. Primary inference was based on multivariable logistic regression; all thresholds, subgroups, mediation, visualization, and matching analyses were exploratory or supportive.

## 3 Results

### 3.1 Study population and baseline metabolic profile

The final analytical cohort included 583 pregnant women, of whom 37 subsequently developed GDM, while 546 did not. Included and excluded participants were broadly comparable in terms of key demographic, anthropometric, and liver metabolic characteristics (**Supplementary Table 1**). Among participants with available outcome data, the proportion of women with GDM was similar between the excluded and included participants [2/27 (7.4%) vs. 37/583 (6.3%); P = 0.688].

The baseline characteristics according to subsequent GDM status are shown in **Table 1**. Women who developed GDM had higher adiposity-related measures before and during early pregnancy, including BMI and eBFP. They also had higher early pregnancy liver metabolic indices, including ALT, HSI, untransformed FLI, and FLI_Ln. Conversely, maternal age, parity, gestational age at assessment, and AST did not differ significantly between the groups. However, the eBFP change rate was numerically higher among women who later developed GDM, although the unadjusted between-group difference was not statistically significant [0.71 ± 1.31 vs. 0.37 ± 0.98; P = 0.122]. This pattern was further evaluated using the covariate-adjusted models. Comparisons of metabolic biomarkers (e.g., glucose, HOMA-IR, free fatty acids, and lipid parameters) by GDM status are provided in **Supplementary Table 2**.

**Table 1:** Baseline and early-pregnancy characteristics of the analytic cohort according to subsequent GDM.

Characteristic	Overall (n = 583)	No GDM (n = 546)	GDM (n = 37)	P value
Demographic and obstetric characteristics				
Age, years	32.1 ± 3.8	32.0 ± 3.8	32.5 ± 3.6	0.423
Parity status, n (%)				0.903
Nulliparous	278 (47.7)	260 (47.6)	18 (48.6)	
Parous	305 (52.3)	286 (52.4)	19 (51.4)	
Gestational age at assessment, weeks	12.4 ± 0.5	12.4 ± 0.5	12.4 ± 0.5	0.490
Adiposity-related measures				
Pre-pregnancy BMI, kg/m²	22.0 ± 3.5	21.8 ± 3.2	25.9 ± 5.2	<0.001
BMI at 10–14 weeks of gestation, kg/m²	22.4 ± 3.5	22.2 ± 3.2	26.7 ± 5.0	<0.001
Pre-pregnancy eBFP, %	28.4 ± 4.3	28.1 ± 3.9	33.2 ± 6.2	<0.001
eBFP at 10–14 weeks of gestation, %	28.9 ± 4.4	28.6 ± 4.0	34.1 ± 6.1	<0.001
eBFP change rate, scaled units	0.39 ± 1.01	0.37 ± 0.98	0.71 ± 1.31	0.122
Hepatic–metabolic measures				
AST, IU/L	17.8 ± 8.1	17.7 ± 8.2	19.9 ± 7.3	0.113
ALT, IU/L	11.0 (8.0–15.0)	11.0 (8.0–14.0)	15.0 (10.0–25.0)	0.002
HIS	30.3 ± 4.7	30.0 ± 4.4	35.8 ± 5.8	<0.001
FLI	11.5 (6.1–22.8)	10.9 (6.0–20.1)	40.2 (24.8–70.9)	<0.001
FLI_Ln	2.5 ± 0.9	2.4 ± 0.9	3.5 ± 0.9	<0.001

Data are presented as mean ± SD, median (IQR), or n (%), as appropriate. P values are unadjusted and were calculated using Student’s t test, the Mann–Whitney U test, the χ² test, or Fisher’s exact test, as appropriate. Pre-pregnancy eBFP was derived from pre-pregnancy BMI and maternal age using the equation 1.20 × BMI + 0.23 × age − 5.4. BMI at 10–14 weeks of gestation was back-calculated from the HSI formula as described in the Methods, and eBFP at 10–14 weeks of gestation was derived from that back-calculated BMI and maternal age. The scaled eBFP change rate was calculated as [(eBFP at 10–14 weeks of gestation − pre-pregnancy eBFP)/gestational age at early-pregnancy assessment] × 10; therefore, a 1-unit increase represents a 0.1 percentage-point/week higher eBFP change rate.

ALT, alanine aminotransferase; AST, aspartate aminotransferase; BMI, body mass index; eBFP, estimated body fat percentage; FLI, fatty liver index; FLI_Ln, natural log-transformed fatty liver index; GDM, gestational diabetes mellitus; HSI, hepatic steatosis index; IQR, interquartile range; SD, standard deviation.

### 3.2 Association between eBFP change rate and subsequent GDM

Although the unadjusted between-group difference in eBFP change rate was not statistically significant (**Table 1**), the adjusted restricted cubic spline model demonstrated a significant association between the eBFP change rate and GDM, with an approximately linear dose-response pattern (P for overall = 0.006; P for nonlinearity = 0.139; **Figure 2A**). The corresponding overall estimate presented in **Figure 3** showed that the eBFP change rate was positively associated with subsequent GDM after adjustment for maternal age, parity, pre-pregnancy BMI (OR per 0.1 percentage-point/week higher eBFP change rate, 1.58; 95% CI, 1.14–2.21; P = 0.006)—the approximately linear pattern supported modeling the eBFP change rate as a continuous exposure in subsequent analyses. Variance inflation factor analysis confirmed that collinearity was not problematic in the primary model: all VIF values were below the conventional threshold of 5 (**Supplementary Table 3**). Adding the eBFP change rate to a baseline model (age, parity, pre-pregnancy BMI) increased the AUC from 0.760 to 0.787 (P = 0.285 DeLong’s test; **Supplementary Figure 2**).

**Figure 2:**
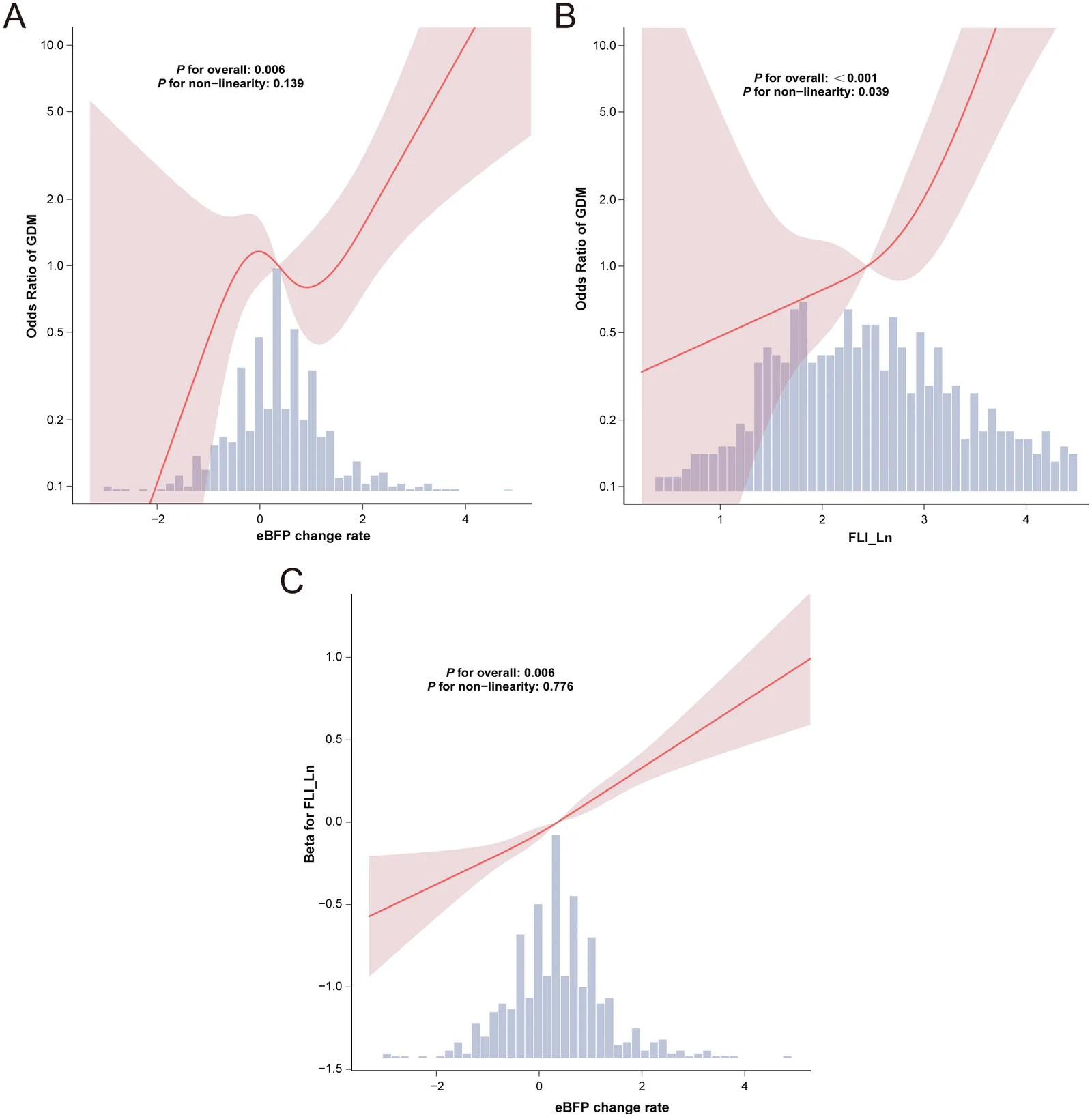
Restricted cubic spline analyses of the associations among early-pregnancy eBFP change rate, FLI_Ln, and subsequent GDM. Restricted cubic spline analyses showing the associations of **(A)** scaled early-pregnancy eBFP change rate with subsequent GDM, **(B)** FLI_Ln with subsequent GDM, and **(C)** scaled eBFP change rate with FLI_Ln. A 1-unit increase in the scaled eBFP change rate corresponds to a 0.1 percentage-point/week higher eBFP change rate. In **(A, B)**, solid lines indicate adjusted odds ratios referenced to the median exposure value; in **(C)**, the solid line indicates the adjusted fitted value of FLI_Ln. Shaded areas indicate 95% confidence intervals, and histograms show exposure distributions. The vertical line in **(B)** marks the data-derived FLI_Ln threshold of 2.90, which should not be interpreted as a validated clinical, diagnostic, or treatment-decision cutoff. Models were adjusted for maternal age, parity, and pre-pregnancy BMI. BMI, body mass index; CI, confidence interval; eBFP, estimated body fat percentage; FLI, fatty liver index; FLI_Ln, natural log-transformed fatty liver index; GDM, gestational diabetes mellitus; OR, odds ratio.

**Figure 3:**
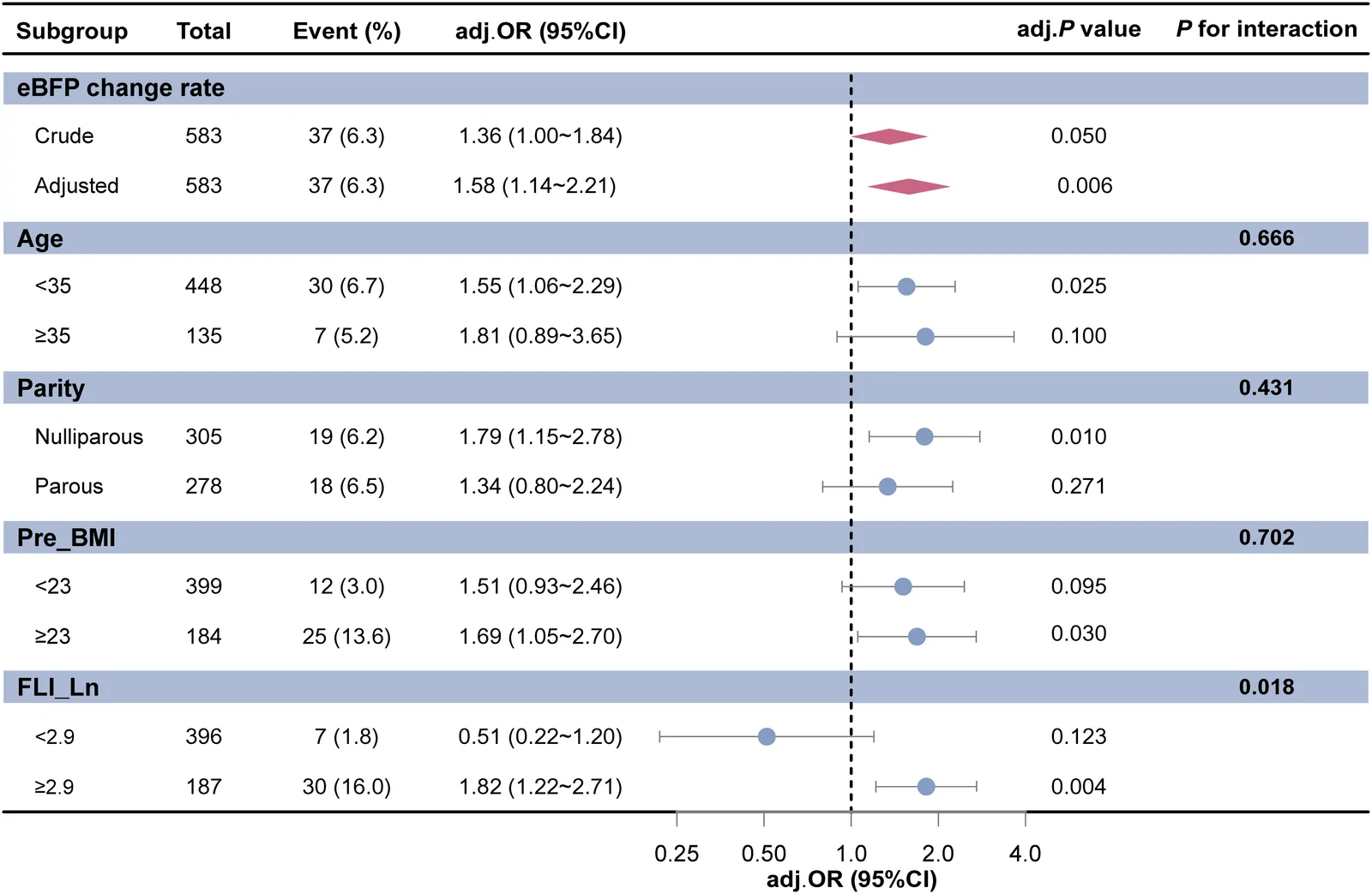
Association of early-pregnancy eBFP change rate with subsequent GDM across predefined subgroups. Forest plot showing crude and adjusted overall estimates and adjusted subgroup estimates for the association between scaled early-pregnancy eBFP change rate and subsequent GDM. ORs and 95% CIs are shown per 1-unit increase in the scaled eBFP change rate, equivalent to a 0.1 percentage-point/week higher eBFP change rate. Subgroups were defined by maternal age (<35 vs. ≥35 years), parity, pre-pregnancy BMI (<23 vs. ≥23 kg/m²), and FLI_Ln status (<2.90 vs. ≥2.90). Models were adjusted for maternal age, parity, and pre-pregnancy BMI, except for the corresponding stratification factor. P for interaction values were derived from multiplicative interaction terms. CI, confidence interval; eBFP, estimated body fat percentage; FLI, fatty liver index; FLI_Ln, natural log-transformed fatty liver index; GDM, gestational diabetes mellitus; OR, odds ratio.

### 3.3 FLI_Ln in relation to GDM and eBFP change rate

FLI_Ln was used in subsequent analyses to improve distributional symmetry because the untransformed FLI was right-skewed (**Supplementary Figure 1**). The adjusted restricted cubic spline model showed that FLI_Ln was significantly associated with subsequent GDM, with evidence of nonlinearity (P for overall = 0.001; P for nonlinearity = 0.039; **Figure 2B**). Segmented regression identified an inflection point at FLI_Ln = 2.90 (95% CI, 2.88–2.93; **Table 2**). Below this value, FLI_Ln was not significantly associated with GDM (OR per 1-unit increase, 0.99; 95% CI, 0.19–5.26; P = 0.992), whereas above this value, higher FLI_Ln was associated with higher odds of GDM (OR per 1-unit increase, 14.06; 95% CI, 3.13–63.22; P < 0.001). This segmented model provided a better fit than the single-line model (likelihood ratio test, P = 0.007). Therefore, FLI_Ln = 2.90 was used as a data-derived threshold for subsequent stratified analyses, but not as a validated clinical diagnostic cutoff point.

**Table 2:** Exploratory segmented association between FLI_Ln and subsequent GDM.

Parameter	No. of participants	Breakpoint or adjusted OR (95% CI)	P value
Estimated breakpoint for FLI_Ln	583	2.90 (2.88–2.93)	—
FLI_Ln < 2.90	396	0.99 (0.19–5.26)	0.992
FLI_Ln ≥ 2.90	187	14.06 (3.13–63.22)	<0.001
Likelihood ratio test	583	—	0.007

Values are from an exploratory segmented logistic regression model assessing the association between continuous FLI_Ln and subsequent gestational diabetes mellitus. The second and third rows show adjusted ORs per 1-unit higher FLI_Ln below and above the estimated breakpoint, respectively. The model was adjusted for maternal age, parity, pre-pregnancy BMI, and gestational age at early-pregnancy assessment. The likelihood ratio test compared the segmented model with a model containing a single linear term for FLI_Ln. The breakpoint at FLI_Ln = 2.90 was data-derived.

CI, confidence interval; FLI, fatty liver index; FLI_Ln, natural log-transformed fatty liver index; OR, odds ratio.

Additionally, the eBFP change rate was positively associated with FLI_Ln values. Restricted cubic spline analysis revealed a significant overall association without any evidence of nonlinearity (P for overall = 0.006; P for nonlinearity = 0.776; **Figure 2C**). These findings provide a rationale for evaluating FLI_Ln as a potential effect modifier of the association between the eBFP change rate and GDM.

### 3.4 The Association between eBFP change rate and GDM was more evident among women with elevated FLI_Ln

Subgroup analyses were conducted to assess whether the association between the eBFP change rate and subsequent GDM differed according to maternal age, parity, pre-pregnancy BMI, or FLI_Ln status (**Figure 3**). No significant interactions were observed between subsequent GDM and maternal age, parity, or pre-pregnancy BMI. In contrast, FLI_Ln status significantly modified the association between the eBFP change rate and GDM. Among women with FLI_Ln ≥ 2.90, a higher eBFP change rate was associated with increased odds of subsequent GDM (OR per 0.1 percentage-point/week higher eBFP change rate, 1.82; 95% CI, 1.22–2.71; P = 0.004). Conversely, among women with FLI_Ln < 2.90, this association was not statistically significant (OR, 0.51; 95% CI, 0.22–1.20; P = 0.123). Thus, the interaction between eBFP change rate and FLI_Ln status was statistically significant (P for interaction = 0.018; **Figure 3**).

Stratified restricted cubic spline analyses further supported this modification pattern (**Figure 4**). Among women with FLI_Ln ≥ 2.90, the eBFP change rate showed a significant overall association with subsequent GDM, without evidence of nonlinearity (P for overall = 0.026; P for nonlinearity = 0.396). By contrast, no significant association was observed among women with FLI_Ln < 2.90 (P for overall = 0.649; P for nonlinearity = 0.522). Therefore, the absence of an association in participants with FLI_Ln <2.90 group should be interpreted as imprecise rather than definitive evidence of no association because only seven GDM events occurred in this stratum. Together, the subgroup and stratified spline analyses indicated that the positive association between eBFP change rate and GDM was concentrated among women with elevated FLI_Ln.

**Figure 4:**
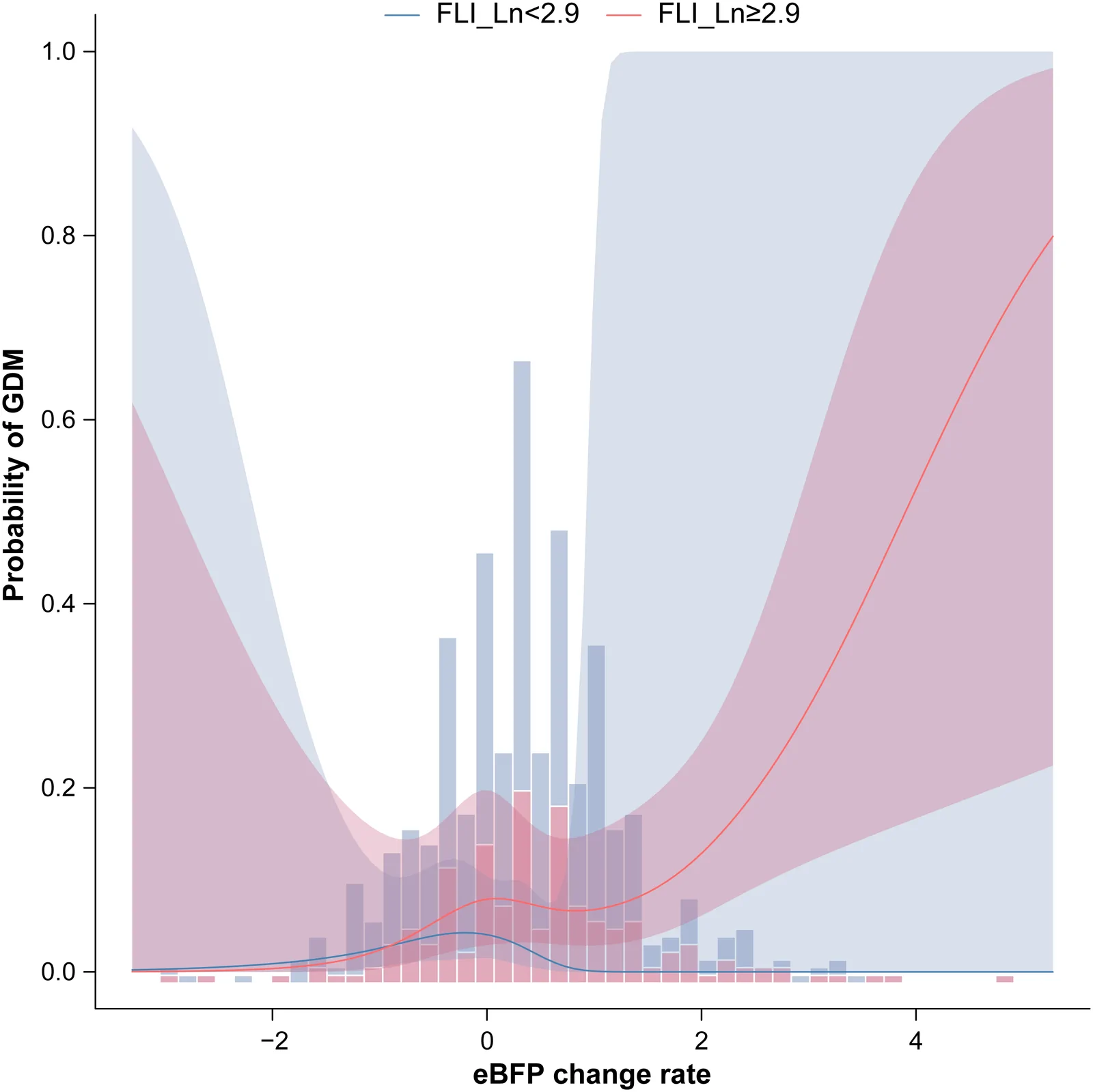
Predicted probability of subsequent GDM by scaled early-pregnancy eBFP change rate, stratified by FLI_Ln status. Restricted cubic spline plots showing the predicted probability of subsequent GDM according to scaled early-pregnancy eBFP change rate, stratified by FLI_Ln status. A 1-unit increase in the scaled eBFP change rate corresponds to a 0.1 percentage-point/week higher eBFP change rate. Curves represent women with FLI_Ln <2.90 and FLI_Ln ≥2.90; shaded areas indicate 95% confidence intervals, and histograms show the distribution of scaled eBFP change rate within each stratum. Models were adjusted for maternal age, parity, and pre-pregnancy BMI. CI, confidence interval; eBFP, estimated body fat percentage; FLI, fatty liver index; FLI_Ln, natural log-transformed fatty liver index; GDM, gestational diabetes mellitus.

### 3.5 Exploratory and sensitivity analyses

#### 3.5.1 Exploratory mediation analysis using elevated FLI_Ln

An exploratory mediation analysis was performed to examine whether elevated FLI_Ln partly explained the association between the eBFP change rate and subsequent GDM (**Figure 5**). An elevated FLI_Ln was defined using a data-derived threshold of 2.90. Higher eBFP change rate was associated with greater odds of elevated FLI_Ln (OR per 0.1 percentage-point/week higher eBFP change rate, 1.89; 95% CI, 1.47–2.42; P < 0.001), and elevated FLI_Ln was associated with higher odds of GDM after adjustment for eBFP change rate and covariates (OR, 4.96; 95% CI, 1.80–13.61; P = 0.002). The indirect effect of elevated FLI_Ln was significant, whereas the direct effect was attenuated after the inclusion of elevated FLI_Ln. The estimated proportion mediated was 12.45%; however, the wide confidence interval indicated considerable uncertainty in the magnitude of mediation (95% CI, 1.60%–74.38%; P = 0.038).

**Figure 5:**
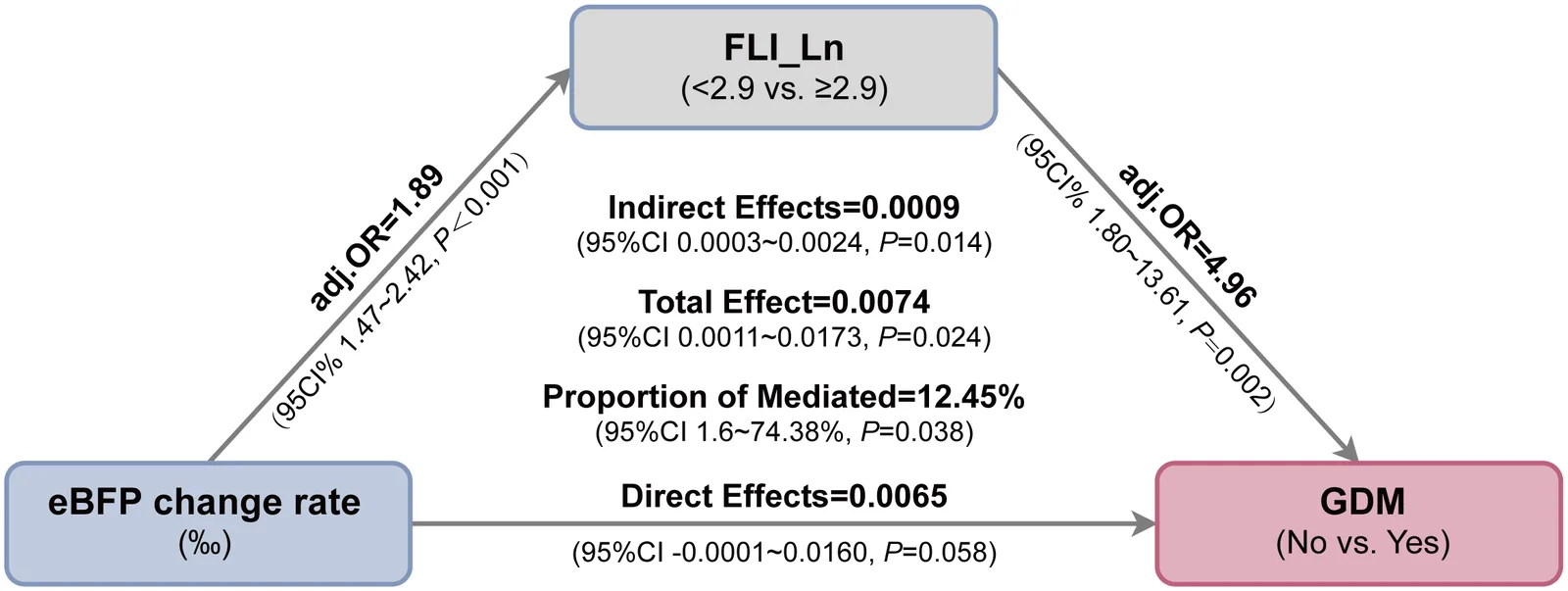
Exploratory mediation analysis using elevated FLI_Ln as the mediator. Exploratory mediation model evaluating elevated FLI_Ln (≥2.90) as a statistical mediator of the association between scaled early-pregnancy eBFP change rate and subsequent GDM. A 1-unit increase in the scaled eBFP change rate corresponds to a 0.1 percentage-point/week higher eBFP change rate. Path labels show adjusted OR with 95% CI where applicable. Values below the mediator box show the total, indirect, and direct effects and the proportion mediated, with corresponding 95% CI and P values. Models were adjusted for maternal age, parity, pre-pregnancy BMI. The FLI_Ln threshold was data-derived. CI, confidence interval; eBFP, estimated body fat percentage; FLI, fatty liver index; FLI_Ln, natural log-transformed fatty liver index; BMI, body mass index; GDM, gestational diabetes mellitus; OR, odds ratio.

#### 3.5.2 Exploratory mediation analysis using continuous FLI_Ln

The mediation analysis was repeated using continuous FLI_Ln as the mediator to assess whether this exploratory pattern depended on dichotomizing FLI_Ln (**Figure 6**). A higher eBFP change rate was associated with higher continuous FLI_Ln (adjusted β, 0.19; 95% CI, 0.15–0.23; P < 0.001), and a continuous FLI_Ln was associated with higher odds of GDM after adjustment for eBFP change rate and covariates (adjusted OR, 5.09; 95% CI, 2.28–11.35; P < 0.001). Thus, the indirect effect of the continuous FLI_Ln remained significant. Although the estimated proportion of mediation was 53.98%, its confidence interval was wide and exceeded 100% (95% CI, 20.11%–183.62%; P = 0.016), indicating that the magnitude of mediation was not reliably quantified.

**Figure 6:**
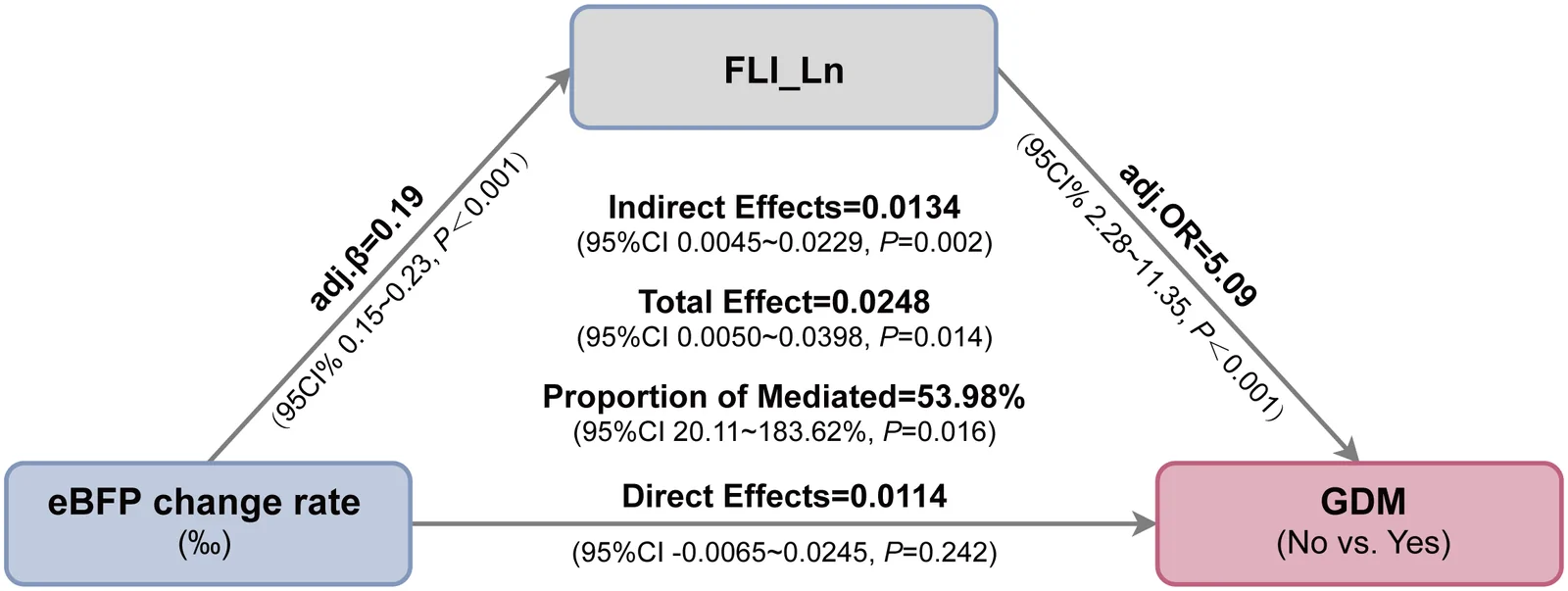
Sensitivity mediation analysis using continuous FLI_Ln as the mediator. Sensitivity mediation model evaluating continuous FLI_Ln as a statistical mediator of the association between scaled early-pregnancy eBFP change rate and subsequent GDM. A 1-unit increase in the scaled eBFP change rate corresponds to a 0.1 percentage-point/week higher eBFP change rate. Path labels show the adjusted β coefficient for the linear association with continuous FLI_Ln and the adjusted OR for the association with subsequent GDM, each with 95% CI. Values below the mediator box show the total, indirect, and direct effects and the proportion mediated, with corresponding 95% CI and P values. Models were adjusted for maternal age, parity, and pre-pregnancy BMI. CI, confidence interval; eBFP, estimated body fat percentage; FLI, fatty liver index; FLI_Ln, natural log-transformed fatty liver index; GDM, gestational diabetes mellitus; OR, odds ratio; β, beta coefficient.

#### 3.5.3 Joint contour plot of eBFP change rate and FLI_Ln

A joint contour plot was generated to visualize the distribution of predicted GDM risk across the eBFP change rate and continuous FLI_Ln (**Figure 7**). The predicted log odds of GDM were highest when both the eBFP change rate and FLI_Ln were high. This descriptive joint risk pattern was consistent with the preceding analyses but should not be interpreted as evidence of biological synergy or causal interaction.

**Figure 7:**
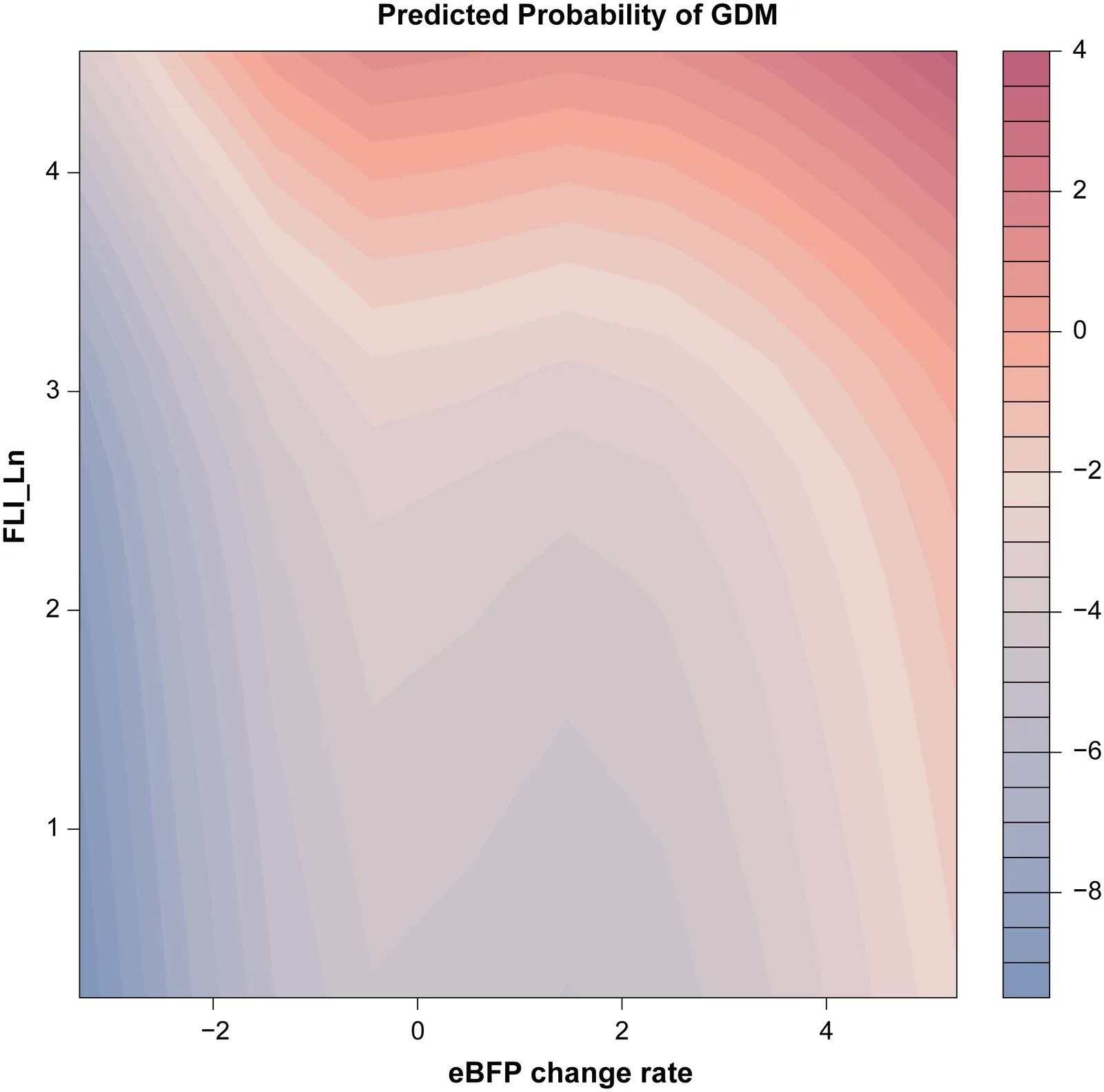
Joint contour plot of predicted GDM probability according to early-pregnancy eBFP change rate and continuous FLI_Ln. Joint contour plot of the predicted probability of subsequent GDM according to early-pregnancy eBFP change rate and continuous FLI_Ln. A 1-unit increase in the scaled eBFP change rate corresponds to a 0.1 percentage-point/week higher eBFP change rate. Warmer colors indicate higher predicted probability of GDM. Models were adjusted for maternal age, parity, and pre-pregnancy BMI. eBFP, estimated body fat percentage; FLI, fatty liver index; FLI_Ln, natural log-transformed fatty liver index; GDM, gestational diabetes mellitus.

#### 3.5.4 Propensity score-matched sensitivity analysis

As a sensitivity analysis, women with FLI_Ln < 2.90 and those with FLI_Ln ≥ 2.90 were matched 1:1 using propensity scores based on maternal age, parity, pre-pregnancy BMI, and gestational age at assessment. The matched cohort included 196 women, with 98 in each FLI_Ln group, and balanced covariates (**Supplementary Figure 3**). In this matched cohort, the GDM incidence was higher among women with FLI_Ln ≥ 2.90 than among those with FLI_Ln < 2.90 [11/98 (11.2%) vs. 2/98 (2.0%); P = 0.010; **Table 3**]. No significant between-group differences were observed in other obstetric or neonatal outcomes (**Table 3**). Given the small number of GDM cases after matching, this analysis was considered supportive rather than confirmatory.

**Table 3:** Pregnancy outcomes in the propensity score–matched cohort according to FLI_Ln status.

Outcome	Overall (n = 196)	FLI_Ln <2.90 (n = 98)	FLI_Ln ≥2.90 (n = 98)	P value
Subsequent GDM, n (%)	13 (6.6)	2 (2.0)	11 (11.2)	0.010
Gestational hypertension or preeclampsia, n (%)	6 (3.1)	4 (4.2)	2 (2.0)	0.442
Gestational age at delivery, weeks	39.0 ± 1.2	38.9 ± 1.2	39.0 ± 1.3	0.496
Preterm birth, n (%)	10 (5.1)	5 (5.1)	5 (5.1)	>0.999
Cesarean delivery, n (%)	83 (42.6)	41 (41.8)	42 (43.3)	0.836
Birth weight, kg	3.2 ± 0.4	3.2 ± 0.4	3.3 ± 0.4	0.100
Birth weight category, n (%)				0.701
Low birth weight, 1.5**–<**2.5 kg	4 (2.0)	3 (3.1)	1 (1.0)	
Normal birth weight, 2.5 kg**–<**4.0 kg	186 (94.9)	92 (93.9)	94 (95.9)	
Macrosomia, **≥**4.0 kg	6 (3.1)	3 (3.1)	3 (3.1)	
Large for gestational age, n (%)	23 (11.7)	10 (10.2)	13 (13.3)	0.506

Values are mean ± SD or n (%), with percentages based on available data. The matched cohort was generated by 1:1 nearest-neighbor propensity score matching using maternal age, parity, pre-pregnancy BMI, and gestational age at assessment. P values compare women with FLI_Ln <2.90 and women with FLI_Ln ≥2.90; the P value for birth weight category represents the overall comparison across categories. Data on gestational hypertension or preeclampsia were missing for 9 participants and data on cesarean delivery for 1 participant. Given the small number of GDM events, results should be interpreted as supportive rather than confirmatory.

BMI, body mass index; FLI, fatty liver index; FLI_Ln, natural log-transformed fatty liver index; GDM, gestational diabetes mellitus; SD, standard deviation.

Collectively, these exploratory analyses supported a pattern in which elevated FLI_Ln was associated with the eBFP change rate and subsequent GDM, without establishing a definitive causal explanatory pathway.

## 4 Discussion

The principal finding of this study was that a faster early pregnancy BMI-derived estimated adiposity change rate was associated with a higher risk of subsequent GDM, particularly among women with an elevated composite liver-related metabolic phenotype, as reflected by FLI_Ln. This association remained evident even after accounting for maternal age, parity, pre-pregnancy BMI, and gestational age at assessment, suggesting that early dynamic adiposity-related changes may carry risk information that is not fully captured by baseline maternal body size. Additionally, we observed a nonlinear association between FLI_Ln and GDM risk, with the eBFP change rate and GDM association being more apparent in women above the data-derived FLI_Ln threshold. Taken together, these findings suggest that early GDM susceptibility may be shaped by the interplay between dynamic adiposity-related change and hepatic metabolic burden, rather than by pre-pregnancy BMI alone (5, 24, 25).

These findings refine the conventional BMI-centered framework for GDM risk assessment by shifting the focus from baseline maternal body size to the tempo of early pregnancy adiposity-related changes. In the present study, the eBFP change rate should be interpreted cautiously as a BMI-derived estimate and not as a direct measure of fat mass gain; nevertheless, it may serve as a pragmatic marker of early adiposity-related dynamics. This interpretation is supported by previous studies linking first-trimester body composition, abdominal adiposity, and early gestational weight-gain patterns with subsequent dysglycemia or GDM risk (24–26). Thus, our study shifts the clinical focus from “how large is maternal body size before pregnancy?” to “How rapidly does adiposity-related status change during early pregnancy?”

Furthermore, our findings indicate that the clinical significance of this dynamic change depends on the concurrent hepatic-metabolic context. FLI_Ln should not be interpreted as a liver-specific biomarker or as a diagnostic surrogate for NAFLD, because the original FLI incorporates BMI, WC, TG, and GGT (12). Instead, FLI_Ln is better understood as a BMI-containing composite hepatic-metabolic phenotype that integrates central adiposity, lipid metabolism, and liver enzyme–related metabolic burden. This interpretation is consistent with previous evidence linking first-trimester ultrasound-defined NAFLD, FLI, and HSI with impaired glucose metabolism and subsequent GDM risk (9, 27, 28). Additionally, the value of FLI_Ln in the present study lies less in its standalone association with GDM than in its ability to define the hepatic-metabolic context in which early BMI-derived adiposity-related changes become more strongly associated with GDM. Specifically, the positive association between eBFP change rate and GDM was more apparent among women above the data-derived FLI_Ln threshold, whereas the estimates below this threshold were limited by a small number of GDM events. These findings suggest that early adiposity-related changes may become metabolically consequential, primarily when they occur in an unfavorable hepatic-metabolic background. Accordingly, FLI_Ln should be viewed as a contextual risk phenotype rather than as evidence of a single hepatic causal pathway. Therefore, our exploratory mediation findings should be interpreted as statistical support for a shared risk pattern, and not as proof of causal mediation (29).

A biologically plausible explanation for these findings is that accelerated early BMI-derived adiposity-related changes may increase metabolic demand, whereas elevated FLI_Ln may reflect a hepatic-metabolic milieu that is less able to buffer that demand. Normal pregnancy involves a transition from early nutrient storage to progressive insulin resistance, and maintenance of maternal euglycemia requires adequate β-cell compensation (30, 31). In contrast, in women with more rapid early adiposity-related dynamics, increased lipid flux and adipose-tissue stress could be accompanied by higher free fatty acid exposure, altered adipokine signaling, and low-grade inflammation, factors that have been implicated in pregnancy-related insulin resistance (32, 33). When superimposed on an elevated composite hepatic–metabolic phenotype, these changes may further aggravate hepatic insulin resistance and dysregulated hepatic glucose–lipid handling, thereby increasing the compensatory burden on β cells (34, 35). In case of insufficient β-cell compensation, this combined adipose–hepatic metabolic stress may become clinically evident as GDM during mid-pregnancy (31, 35). Notably, this interpretation should be considered biologically plausible rather than mechanistically proven, as our study did not directly assess insulin sensitivity, insulin secretion, free fatty acids, adipokines, inflammatory markers, or quantitative liver fat.

mTORC1 signaling may serve as a convergent molecular node linking adiposity-related dynamics, hepatic metabolic status, and subsequent dysglycemia. mTORC1 integrates nutrient and insulin signals to regulate β-cell function, hepatic lipid metabolism, and systemic insulin sensitivity (36, 37). mTORC1 directly phosphorylates PDX1 at S61 to promote β-cell insulin expression; under chronic nutrient excess, the same signal can drive hyperinsulinemia, obesity, and hepatic steatosis (36). In early pregnancy, accelerated adiposity-related change may increase mTORC1 flux, physiologically supporting β-cell expansion (37); superimposed on an elevated hepatic-metabolic phenotype, chronic mTORC1 overactivation could exacerbate insulin resistance and lipid accumulation, and if β-cell compensation fails, dysglycemia may ensue (36, 38). This framework is biologically plausible but remains speculative in the absence of direct mTOR pathway measurements.

Nevertheless, this combined framework may have potential utility for early risk enrichment before routine OGTT. Current standards generally recommend GDM screening at 24–28 weeks of gestation in individuals without previously diagnosed diabetes. However, the present findings raise the possibility that a metabolically vulnerable subgroup may be recognizable during the first trimester (4, 39). In this regard, this adiposity–hepatic framework may help identify women who could be considered for earlier lifestyle counseling, individualized nutritional guidance, gestational weight-gain monitoring, or closer glycemic surveillance in future validated care pathways, given that pre-pregnancy BMI, early-pregnancy BMI-derived eBFP change rate, and FLI_Ln-related components can be obtained through anthropometric assessment and routine or low-burden biochemical testing in many clinical settings (4, 39, 40). Notably, this framework should be viewed as risk enrichment rather than early diagnosis and should not replace standard OGTT-based screening. Although the coherence of the observed pattern was supported by convergent results across the dose–response assessment, threshold-based stratification, interaction analyses, joint-pattern visualization, propensity score–matched comparisons, and exploratory mediation analyses, these analyses are hypothesis-generating. Consequently, future studies are required to evaluate whether adding eBFP change rate and FLI_Ln to established GDM risk models improves discrimination, calibration, clinical net benefit, and risk-guided intervention outcomes before clinical trials application.

Despite the positive findings, this study has some limitations that should be considered when interpreting the findings. First, this study was a secondary analysis of a publicly available prospective cohort; the hypotheses and exposure metrics were not pre-specified, and unmeasured confounders (family history of diabetes, smoking, socioeconomic status, dietary data) could not be accounted for. Second, early-pregnancy BMI was derived from HSI rather than measured directly; rounding of the deposited values may introduce minor imprecision (≤ 0.1 kg/m²). Third, the FLI_Ln threshold identified in this study was derived from publicly available data and should not be interpreted as a clinically validated diagnostic cutoff value. Fourth, the mediation analyses were exploratory and should be considered as a statistical decomposition of an observed association rather than evidence of a causal pathway (29). However, VIF analysis confirmed acceptable collinearity (all VIF < 5), the eBFP change rate, FLI_Ln, and pre-pregnancy BMI share BMI-related components and are not fully independent constructs. Fifth, the mechanistic interpretation is limited because insulin sensitivity, insulin secretion, free fatty acids, adipokines, inflammatory markers, and quantitative hepatic fat were not directly assessed. Sixth, preconception FLI data were unavailable, which limits our ability to disentangle pre-existing hepatic-metabolic burden from early-gestational change. Future studies with preconception measures could help clarify this temporal relationship. Finally, the study population consisted of pregnant Korean women from a specific cohort, and generalizability to other racial or ethnic groups, BMI distributions, GDM diagnostic strategies, and healthcare systems should be approached with caution. Nevertheless, our findings support the notion that early GDM susceptibility may be better captured by the interplay between dynamic adiposity-related changes and hepatic metabolic burden than by pre-pregnancy BMI alone. Consequently, future studies incorporating standardized metabolic phenotyping, quantitative liver fat assessment, indices of insulin sensitivity, and β-cell function, as well as external validation cohorts, are needed to confirm this early adipose–hepatic metabolic susceptibility model.

## 5 Conclusion

In this secondary analysis of a prospective Korean pregnancy cohort, a higher early-pregnancy eBFP change rate was associated with a higher risk of subsequent GDM, particularly among women with elevated FLI_Ln. These findings suggest that early adiposity-related changes may be more clinically informative when considered alongside hepatic metabolic burden, rather than pre-pregnancy BMI alone. However, this approach is intended for risk enrichment, not early diagnosis, and should not replace standard OGTT-based screening. Future studies should validate these findings in external cohorts and determine whether incorporating eBFP change rate and FLI_Ln into early pregnancy risk assessment can improve GDM prediction and help guide earlier lifestyle counseling, gestational weight-gain monitoring, or closer glycemic surveillance.

## Data Availability

The raw data supporting the conclusions of this article will be made available by the authors, without undue reservation.
