# Supplementary material for: Early-pregnancy BMI-derived adiposity change rate and gestational diabetes mellitus risk: modification by a fatty liver index–defined hepatic-metabolic phenotype
Source: Front Endocrinol (Lausanne). 2026 Sep 15;17:1889628. doi: 10.3389/fendo.2026.1889628 (PMC13619510; doi:10.3389/fendo.2026.1889628)
Supplement: Supplementary file 1 [file Supplementaryfile1.docx]

Supplementary Figure S1. Distribution of fatty liver index (FLI) before and after natural log transformation.

Figure S1 legend: (A) Rank-ordered values of FLI, with an inset box plot. (B) Histogram of FLI with an overlaid density curve, illustrating a right-skewed distribution. (C) Rank-ordered values of FLI_Ln, with an inset box plot. (D) Histogram of FLI_Ln with an overlaid density curve, showing reduced skewness and improved distributional symmetry compared with FLI.

Supplementary Table S1. Comparison of characteristics between included and excluded participants.

| **Characteristic** | **Overall**  **(n = 623)** | **Excluded**  **(n = 40)** | **Included**  **(n = 583)** | **P value** |
| --- | --- | --- | --- | --- |
| Age, years | 32.1 ± 3.9 | 32.2 ± 5.2 | 32.1 ± 3.8 | 0.825 |
| Parity status, no. (%) |  |  |  | 0.555 |
| Parous | 324 (52.0) | 19 (47.5) | 305 (52.3) |  |
| Nulliparous | 299 (48.0) | 21 (52.5) | 278 (47.7) |  |
| Pre-pregnancy BMI, kg/m² | 22.1 ± 3.5 | 22.7 ± 3.5 | 22.0 ± 3.5 | 0.274 |
| Gestational age at assessment, weeks | 12.4 ± 0.5 | 12.4 ± 0.5 | 12.4 ± 0.5 | 0.956 |
| AST, IU/L | 17.8 ± 8.1 | 18.0 ± 6.3 | 17.8 ± 8.1 | 0.934 |
| ALT, IU/L | 11.0 (8.0–15.0) | 11.0 (7.2–16.2) | 11.0 (8.0–15.0) | 0.906 |
| FLI | 11.5 (6.1–22.8) | 15.5 (9.4–36.5) | 11.4 (6.0–22.8) | 0.233 |
| HSI | 30.4 ± 4.8 | 30.7 ± 5.3 | 30.3 ± 4.7 | 0.758 |
| GDM, n/available n (%) | 39/610 (6.4) | 2/27 (7.4) | 37/583 (6.3) | 0.688 |

Note: Continuous variables are presented as mean ± SD or median (IQR), as appropriate, and categorical variables as n (%), unless otherwise indicated. Summaries are based on available data for each variable. P values compare excluded and included participants and were calculated using Student’s t test, the Mann–Whitney U test, the χ² test, or Fisher’s exact test, as appropriate. GDM status was unavailable for 13 participants; therefore, the GDM row is presented as n/available n (%), with denominators reflecting available outcome data: overall, n = 610; excluded participants, n = 27; included participants, n = 583.
Abbreviations: ALT, alanine aminotransferase; AST, aspartate aminotransferase; BMI, body mass index; FLI, fatty liver index; GDM, gestational diabetes mellitus; HSI, hepatic steatosis index; IQR, interquartile range; SD, standard deviation.

Supplementary Table S2. Additional early-pregnancy clinical and metabolic characteristics of the analytic cohort according to subsequent GDM status.

| **Characteristic** | **Overall**  **(n = 583)** | **No GDM**  **(n = 546)** | **GDM**  **(n = 37)** | **P value** |
| --- | --- | --- | --- | --- |
| NAFLD, no. (%) |  |  |  | < 0.001 |
| No | 472 (81.0) | 456 (83.5) | 16 (43.2) |  |
| Yes | 111 (19.0) | 90 (16.5) | 21 (56.8) |  |
| GGT, U/l | 14.1 ± 8.7 | 13.8 ± 8.4 | 18.8 ± 11.0 | < 0.001 |
| Cholesterol, mmol/l | 173.0 ± 27.1 | 172.4 ± 26.9 | 180.9 ± 29.6 | 0.066 |
| Triacylglycerol, mmol/l | 119.1 ± 47.6 | 115.2 ± 41.7 | 176.2 ± 81.9 | < 0.001 |
| HDL-cholesterol, mmol/l | 64.9 ± 13.5 | 65.3 ± 13.3 | 59.3 ± 16.4 | 0.009 |
| LDL cholesterol, mmol/l | 84.1 ± 21.6 | 84.1 ± 21.1 | 84.3 ± 28.4 | 0.952 |
| Fasting glucose, mg/dl | 76.9 ± 9.7 | 76.4 ± 9.0 | 84.6 ± 15.2 | < 0.001 |
| Fasting insulin, μIU/ml | 8.4 (5.3, 11.6) | 8.1 (5.3, 11.2) | 15.3 (9.1, 22.5) | < 0.001 |
| Free fatty acids, μEq/l | 650.6 ± 276.3 | 642.4 ± 269.3 | 772.0 ± 346.7 | 0.006 |
| Adiponectin, ng/l | 5036.4 (2824.8, 8047.1) | 5392.0 (3129.5, 8238.2) | 1939.7 (1297.8, 2831.6) | < 0.001 |
| HOMA-IR | 1.5 (1.0, 2.3) | 1.5 (0.9, 2.2) | 2.9 (1.7, 4.7) | < 0.001 |

Note: Continuous variables are presented as mean ± SD or median (IQR), as appropriate, and categorical variables as n (%). P values compare women without and with subsequent GDM and were calculated using Student's t test, the Mann-Whitney U test, the χ² test, or Fisher's exact test, as appropriate.
Abbreviations: NAFLD, nonalcoholic fatty liver disease; GGT, gamma-glutamyl transferase; HOMA-IR, homeostatic model assessment of insulin resistance

Supplementary Table S3. Variance inflation factor analysis for the collinearity diagnostic model.

| **Variable** | **VIF** |
| --- | --- |
| Pre-pregnancy BMI | 3.44 |
| FLI_Ln | 3.23 |
| eBFP change rate | 1.25 |
| Parity | 1.12 |
| Maternal age | 1.10 |
| Gestational age at assessment | 1.02 |

Note: Variance inflation factors were calculated in a collinearity diagnostic model including the scaled eBFP change rate, FLI_Ln, pre-pregnancy BMI, maternal age, parity, and gestational age at assessment. All VIF values were below 5, indicating no evidence of problematic multicollinearity.
Abbreviations: BMI, body mass index; eBFP, estimated body fat percentage; FLI, fatty liver index; FLI_Ln, natural log-transformed fatty liver index; VIF, variance inflation factor.

Supplementary Figure S2. Receiver operating characteristic (ROC) curves for GDM prediction.

Figure S2 legend: ROC curves comparing two models for predicting subsequent GDM. Model 1: maternal age, parity, and pre-pregnancy BMI. Model 2: Model 1 plus the eBFP change rate. AUC values with 95% confidence intervals and the P value from DeLong's test are shown in the inset.
Abbreviations: AUC, area under the curve; BMI, body mass index; eBFP, estimated body fat percentage; GDM, gestational diabetes mellitus; ROC, receiver operating characteristic.

Supplementary Figure S3. Covariate balance before and after propensity score matching by FLI_Ln status.

Figure S3 legend: SMDs for maternal age, parity, pre-pregnancy BMI, and gestational age at assessment before and after 1:1 propensity score matching in women with FLI_Ln <2.90 vs ≥2.90. Hollow circles indicate the unmatched cohort and solid circles the matched cohort. The vertical reference line at an absolute SMD of 0.10 indicates the prespecified threshold for acceptable covariate balance. After matching, all covariates had absolute SMDs <0.10.
Abbreviations: BMI, body mass index; FLI, fatty liver index; FLI_Ln, natural log-transformed fatty liver index; PSM, propensity score matching; SMD, standardized mean difference.

.
